# Repetitive Transcranial Magnetic Stimulation (rTMS) in Mild Cognitive Impairment: Effects on Cognitive Functions—A Systematic Review

**DOI:** 10.3390/jcm12196190

**Published:** 2023-09-25

**Authors:** Minoo Sharbafshaaer, Ilaria Gigi, Luigi Lavorgna, Sabrina Esposito, Simona Bonavita, Gioacchino Tedeschi, Fabrizio Esposito, Francesca Trojsi

**Affiliations:** 1 MRI Research Center, Department of Advanced Medical and Surgical Sciences, Università degli Studi della Campania “Luigi Vanvitelli”, 80138 Naples, Italy; minoo.sharbafshaaer@unicampania.it (M.S.); ilaria.gigi@unicampania.it (I.G.); simona.bonavita@unicampania.it (S.B.); gioacchino.tedeschi@unicampania.it (G.T.); fabrizio.esposito@unicampania.it (F.E.); francesca.trojsi@unicampania.it (F.T.); 2 First Division of Neurology, Università degli Studi della Campania “Luigi Vanvitelli”, 80138 Naples, Italy; sabrina.esposito1@unicampania.it

**Keywords:** mild cognitive impairment, repetitive transcranial magnetic stimulation, EEG, functional magnetic resonance imaging, neuroplasticity

## Abstract

Repetitive transcranial magnetic stimulation (rTMS) is a non-invasive brain stimulation technique also used as a non-pharmacological intervention against cognitive impairment. The purpose of the present review was to summarize what is currently known about the effectiveness of rTMS intervention on different cognitive domains in patients with mild cognitive impairment (MCI) and to address potential neuromodulation approaches in combination with electroencephalography (EEG) and neuroimaging, especially functional magnetic resonance imaging (fMRI). In this systematic review, we consulted three main databases (PubMed, Science Direct, and Scopus), and Google Scholar was selected for the gray literature search. The PRISMA flowchart drove the studies’ inclusion. The selection process ensured that only high-quality studies were included; after removing duplicate papers, explicit ratings were given based on the quality classification as high (A), moderate (B), or low (C), considering factors such as risks of bias, inaccuracies, inconsistencies, lack of direction, and publication bias. Seven full-text articles fulfilled the stated inclusion, reporting five double-blind, randomized, sham-controlled studies, a case study, and a randomized crossover trial. The results of the reviewed studies suggested that rTMS in MCI patients is safe and effective for enhancing cognitive functions, thus making it a potential therapeutic approach for MCI patients. Changes in functional connectivity within the default mode network (DMN) after targeted rTMS could represent a valuable indicator of treatment response. Finally, high-frequency rTMS over the dorsolateral prefrontal cortex (DLPFC) has been shown to significantly enhance cognitive functions, such as executive performance, together with the increase of functional connectivity within frontoparietal networks. The main limitations were the number of included studies and the exclusion of studies using intermittent theta-burst stimulation, used in studies on Alzheimer’s disease. Therefore, neuroimaging techniques in combination with rTMS have been shown to be useful for future network-based, fMRI-guided therapeutic approaches.

## 1. Introduction

The size of the elderly population has been increasing worldwide. Moreover, mild cognitive impairment (MCI) affects 10–15% of the population over the age of 65 [1]. It is an intermediate state between normal cognition and dementia [2,3], with essentially preserved functional abilities [4]. In the elderly, the spectrum of cognitive decline ranges from what can be classified as normal cognitive decline with aging to subjective cognitive impairment, MCI, and dementia [5].

MCI is associated with an increased risk of developing dementia [6,7]. Particularly, MCI is a prodromal stage of dementia, characterized by subjective cognitive deficits and objective memory impairment without impairment in daily activity, since memory deficits are the clinical hallmark and the central characteristic of MCI [8]. Thus, given the key criterion that memory, among all cognitive domains, must be impaired, evidence of cognitive decline in one or more cognitive domains is obtained from patients’ reports, although the use of electroencephalography (EEG) and neuroimaging techniques has also contributed to the documentation of individuals at increased risk for dementia and Alzheimer’s disease (AD) [9,10,11,12,13]. Notably, patients with MCI with or without memory deficits might progress to AD, and this progression might also be tracked by radiological biomarkers such as functional imaging and structural MRI measures [14,15]. Among functional imaging approaches, resting-state functional MRI (rs-fMRI) and/or EEG have been used to investigate the functional connectivity (FC) properties of large-scale brain networks in both healthy [16] and MCI subjects [17]. During the last two decades, transcranial magnetic stimulation (TMS) has assumed a prominent role in the functional evaluation and modulation of cortical circuits in MCI [6], because of its capability of testing specific neurotransmitter systems or cortical connections. Moreover, repetitive TMS (rTMS), one of the non-invasive brain stimulation (NIBS) methods, can induce a prolonged modulation of cortical excitability by inducing the plastic properties of cortical synapses [7,17,18].

By selectively interfering with regionally specific cortical processing, rTMS can be used to draw causal links between brain regions and specific behaviors. If stimulating a cortical area significantly affects task performance related to appropriate control conditions, this means that the stimulated area is necessary to perform the task normally [19]. The present tool is a non-invasive, safe, and painless procedure to activate or modulate cortical targets in the central nervous system [20]. Therefore, the time has come to develop appropriate recommendations to inform the use of rTMS in clinical practice, which has been shown to be well tolerated [21]. The electromagnetic field of TMS permeates the scalp and the skull, develops an electric field in the brain tissue, and enables non-invasive activation of the cerebral cortex [22,23]. It is worth mentioning that high-frequency rTMS with a series pulse causes post-stimulation and neurochemical changes which are associated with the increase of synaptic connectivity [24]. There is considerable evidence that the mechanisms of substantial rTMS after-effects resemble long-term potentiation (LTP) [25]. Moreover, this method can affect brain circuit excitability or plastic changes, influencing the expression levels of various receptors and other neuromodulators [26,27]. Particularly, repetitive trains of stimulation may activate, inhibit, or otherwise interfere with the activity of neuronal cortical networks, but this also depends on stimulus frequency and intensity [28] and can modulate cortical excitability after the period of stimulation itself [29,30,31]. rTMS can also modulate the functions of disorganized brain circuits, especially in cognitive impairment [32], albeit it is usually applied over the left or bilateral dorsolateral prefrontal cortex (DLPFC). The treatment response mechanism was therefore supposed to be based on modulations in functional networks, particularly the meso-cortico-limbic reward circuit [33], and the critical role of the left and right DLPFC in proactive and reactive cognitive control has been widely recognized [34,35]. As we gain a better understanding of how rTMS affects different layers of the brain, there will also be significant insights into the effects on synaptic plasticity.

Among the techniques used to measure the effects of rTMS on brain FC, EEG is a neurophysiologic technique used for evaluating and capturing brain activity with high temporal resolution (in the range of units or tens of milliseconds) and no notable adverse effects [36,37,38]. For instance, by means of EEG measurements and EEG-derived metrics, MCI has been related to alterations in widespread interhemispheric and intrahemispheric connectivity as compared to aging [39]. Regarding the concurrent use of EEG and TMS, EEG has been recognized as an appropriate screening method for investigating neural connectivity properties within targeted functional networks [40]. Moreover, rTMS effects on brain plasticity may be elucidated through EEG monitoring [41,42]. Particularly, the rTMS stimulation was shown to affect the spectral power of EEG signals in the alpha band and the phase synchrony between alpha and gamma rhythms [38,41]. Therefore, changes in cognitive performance could be directly linked to the changes in endogenous task-related oscillatory dynamics and not just to the widespread changes in neural activity at the flicker frequency, oscillatory power, and inter-trial coherence at the driving frequency [43]. As the EEG time course after rTMS could also be related to the time course of the ongoing cognitive processes, EEG recordings could be considered an important tool to measure the effects of rTMS on cognitive performance [44,45].

High-frequency and low-frequency rTMS effects have also been demonstrated with fMRI. rTMS has been shown to affect intrinsic brain FC, as measured via rs-fMRI [45,46]. Combining rTMS with functional brain imaging may provide an indication of how localized changes in neural excitability influence network-wide activity and, thereby, can be used to reveal causal relationships between brain areas [47]. Using rs-fMRI to visualize brain networks based on correlated fluctuations in blood oxygenation, the efficacy of different DLPFC rTMS sites has been related to their effects on the subgenual cingulate cortex, which can eventually be selected as an additional or alternative rTMS site besides the DLPFC [48,49]. However, it is challenging to combine rTMS and fMRI to provide important information for neurocognitive models of cognitive dysfunction. In fact, an rTMS-fMRI study revealed that rTMS can affect blood oxygenation level-dependent (BOLD) signals, not only at the stimulation site but also in remote brain structures, which are variably interconnected between each other, also eliciting changes in regional cerebral blood flow (rCBF) [50]. The BOLD signal changes have also been found to be associated with the different conditions of a cognitive paradigm [51,52,53,54]. Therefore, the combination of rTMS and fMRI provides a strategy for controlling neural activity and testing causal theories, with clear downstream potential for clinical applications of rTMS in neurological diseases. For example, network-based fMRI-guided TMS protocols have been proposed to design personalized treatments for MCI [17,55].

As for the disruption of FC networks in MCI, the default mode network (DMN) is the most affected network in MCI, showing topographical overlap with amyloid pathology, especially in the posterior cingulate cortex and in the precuneus [45]. Moreover, several neurophysiological studies have highlighted that, since the earliest stages of cognitive impairment, cortical plasticity in the DLPFC may also be impaired, with a loss of physiological beta-gamma oscillatory response [55]. The reduction of metabolism in the DLPFC has also been considered a specific alteration during the progressive course of MCI [56]. The DLPFC, which anatomically corresponds to Brodmann areas 9 and 46, is a key part of the executive control network (ECN) and of the frontoparietal network (FPN) [57], acting as a modulator of cognitive functions such as attention, working memory, and executive function [58].

In recent years, rTMS has been extensively studied to assess its potential to modulate cognitive function in MCI, and we have sought to pay particular attention to several methodological aspects of these studies. Indeed, there is considerable heterogeneity among the various rTMS treatment protocols reported in the literature for cognitive enhancement in MCI, including, e.g., various combinations of stimulation location, pulse rate, stimulation intensity, number of stimulations delivered, and number of treatment sessions.

In this systematic review, we aimed to overview the effects of rTMS on cognitive outcomes in cohorts of MCI patients targeting the DLPFC in the light of EEG and fMRI findings used in some studies for targeting and monitoring the effects of rTMS on brain FC. To the best of our knowledge, this is the first systematic review with the aim of overviewing the impact of rTMS on cognitive and neuroimaging findings in MCI patients.

## 2. Methods

### 2.1. Study Focus

Our systematic review was conducted on the preferred reporting investigations that have been published from 2018 to 2023, since the topics of interest have been mostly addressed in recent years. The literature search was performed according to the Preferred Reporting Items for Systematic Reviews and Meta-Analyses (PRISMA) guidelines for 2020 [59].

### 2.2. Inclusion and Exclusion Criteria

We included studies that fulfilled all the following criteria: (1) clinical population of patients previously diagnosed with amnestic MCI (aMCI) and non-amnestic MCI (naMCI) [60]; (2) rTMS was the only intervention being investigated without any other behavioral treatment associated; (3) cognitive functions and/or functional MRI or EEG were measured; (4) parallel or cross-over design that utilized active rTMS and a sham-controlled group; and (5) articles written in English. Studies identified through database searches were initially screened based on their title and abstracts. They were subsequently excluded if it was clear from the title or abstract that the study was not relevant or did not meet the inclusion criteria. Finally, studies were excluded if they were conference abstracts/papers.

### 2.3. Search Strategy

We searched three main databases: PubMed, Science Direct, and Scopus English language full-text citation index, through May 2023. In addition, Google Scholar was selected for the gray literature search.

The keywords used for the database searches were “mild cognitive impairment”, “repetitive transcranial magnetic stimulation”, and “rTMS”. Additionally, we searched reference lists of previous reviews on rTMS in MCI (Chou YH et al., 2020; Chu CS et al., 2021; Jiang et al., 2021) [61,62,63] to identify additional relevant articles. A new review article needed to overview the effects of rTMS on cognitive outcomes in cohorts of MCI patients targeting the DLPFC, also in light of EEG and fMRI findings.

### 2.4. Quality Assessment, Study Screening, and Risk of Bias

The selection process ensured that only high-quality and original studies were included, providing a detailed overview of the effects of rTMS on cognitive outcomes in cohorts of MCI patients. The article list’s titles and abstracts were reviewed independently by two authors (MS and FT), and they read the entirety of all recognized full-text articles. After removing duplicate papers and articles that met the exclusion criteria, all remaining articles were examined by three independent writers (IG, LL, and FE), and any disagreements were addressed by consensus. To assess the certainty of the evidence for each outcome of interest, explicit ratings are given based on the quality classification as high (A), moderate (B), or low (C), considering factors such as risks of bias, inaccuracies, inconsistencies, lack of direction, and publication bias. In cases where there was no direct evidence but plausibility or clinical experience with indirect evidence, the panel made a consensus decision labeled “Expert Opinion”.

### 2.5. Data Items

Data extraction included publication details, patient characteristics, and study design. The collected data were classified into three categories: (1) demographic data; (2) intervention details (rTMS stimulation); and (3) cognitive and RS-fMRI/EEG findings.

## 3. Results

### 3.1. Study Selection

**Figure 1:**
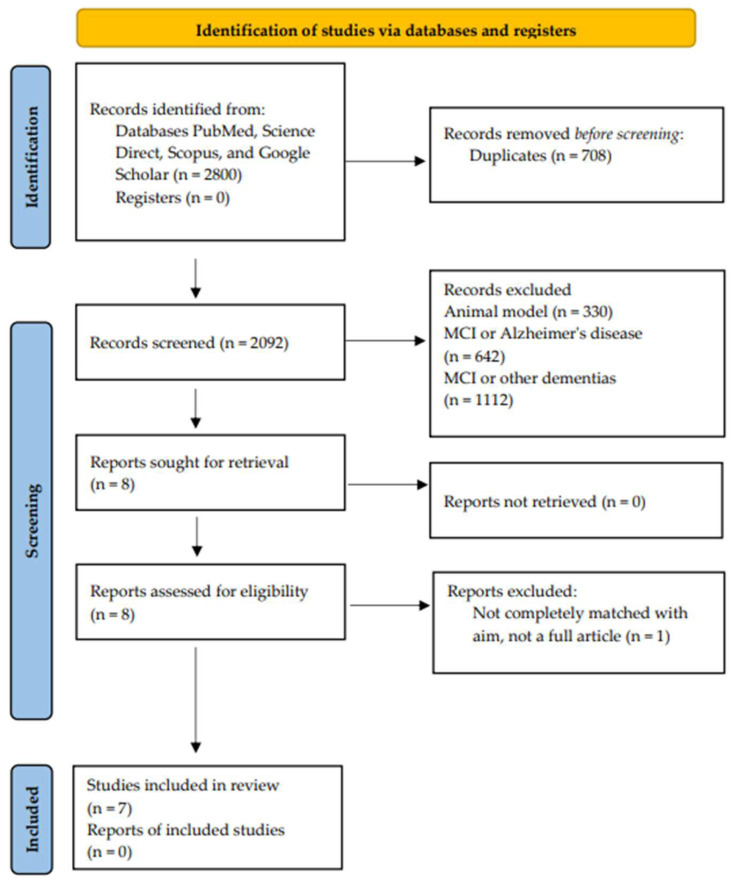
PRISMA flowchart describing study screening and inclusion.

The results of the initial database search were 2800 manuscripts. Duplicate records (n = 708) were eliminated, and records were arranged with titles, abstracts, and original reports (n = 2092). Following a full-text screening, the main exclusion factors were animal research and cohorts of patients with MCI or Alzheimer’s disease/other dementias (n = 2084), not completely matched with aim and not a full article (n = 1). Seven full-text papers that fulfilled the predefined inclusion and exclusion criteria were included in the final synthesis. The study’s inclusion is described in the PRISMA flowchart (Figure 1).

### 3.2. Studies’ Characteristics and Patient Demographics

Basic publication details and patient demographics are presented in Table 1. The included studies [64,65,66,67,68,69] were published between 2018 and May 2023. The smallest study included 3 patients, and the largest included 66 patients. Study designs consisted of five double-blind, randomized, sham-controlled studies [58,64,66,67,69], a case study [65], and a randomized crossover trial (i.e., 22 patients divided into two groups: 11 in group A—sham-active, and 11 in group B—active-sham [68] (Table 1).

### 3.3. Cognitive and Neuroimaging Findings after rTMS Stimulation

Study groups, intervention, stimulation protocols, cognitive/neuroimaging findings, and ORs (95% CIs), with the main results from the 7 selected articles, are shown in Table 2 [64,65,66,67,68,69]. As for rTMS effects on general cognitive outcome, Durand et al. [65] found that all three treated MCI patients improved their cognition and overall clinical state scores, but there was no discernible improvement in their depressive symptoms. Also, Taylor et al. [67] and Roque Roque et al. [68] showed that the groups receiving rTMS stimulation in the DLPFC had improved global cognitive function, in association with the improvement of memory, language, visuoconstructional, processing/executive control, and mood performances. As for rTMS effects on specific cognitive outcomes, Padala et al. [64] revealed that the rTMS group showed more improvement in apathy, executive function, and clinical overall impression, as well as Esposito et al. [69], who showed considerably improved semantic fluency and visuospatial abilities in the treated group. Finally, as for rTMS effects on neuroimaging outcomes, Cui et al. [66] demonstrated significant FC changes within the DMN (between the posterior cingulate gyrus and the right fusiform gyrus), as well as improvement in neuropsychological performance (auditory verbal learning and recall recognition) in the rTMS group. Yuan et al. [58] described significantly increased global cognition in association with changes in amplitude low-frequency fluctuation (ALFF) in the stimulated group compared to the sham group: the ALFF values in the right superior frontal gyrus were considerably lowered in the rTMS group, whereas the ALFF values in the right inferior frontal gyrus, triangular section of the inferior frontal gyrus, right precuneus, left angular gyrus, and right supramarginal gyrus significantly increased. Moreover, Esposito et al. [69] revealed higher FC in the salience network of the rTMS group at the short-term timepoint (i.e., after 1 month from rTMS), while, at the long-term timepoint (i.e., after six months from rTMS), a significant increase of FC in the left frontoparietal network was revealed in the rTMS group.

Of the 7 selected studies, 6 studies [64,66,67,68,69] were assessed as having high quality (A) and low risk of bias, while 1 study [65] was assessed as having moderate quality (B) and potential risk of bias due to the open-label design (three case studies).

## 4. Discussion

This systematic review revealed that rTMS with low-/high-frequency stimulation in the left/right or bilateral DLPFC might have a positive effect on cognition (i.e., executive, memory, language, and visuospatial functions) and behavior abnormalities (i.e., apathy) [64,65,66,67,68,69] in MCI patients. Inversely, conflicting results are reported regarding the effects of rTMS on depression [65,66,67,68,69] in MCI patients, while no evidence has been reported regarding the effects of rTMS on their functional status (i.e., IADL) and on the caregiver burden (i.e., ZBS) [64]. On the other hand, data from fMRI have been shown to be informative for understanding the consequences of rTMS after the stimulation protocol [66,67,69] and at short- and long-term timepoints during follow-up [69]. Therefore, the combination of functional neuroimaging techniques, besides neuropsychological assessment, for assessing and monitoring rTMS effects would be preferred.

Reviewed studies suggest that rTMS in MCI patients is safe and effective for enhancing cognitive function, thus making it a potential therapeutic approach for MCI patients. Our findings resemble evidence from rTMS studies performed in patients with vascular disease; in particular, rTMS has been reported to increase the impaired hemisphere’s excitability and/or modulate the unaffected hemisphere’s activity [53,70]. A study performed in nondemented vascular cognitive impairment (vascular cognitive impairment-no dementia, VCI-ND) revealed that high-frequency rTMS on the ipsilesional DLPFC might exert an immediate effect on cognition by inducing the anti-inflammatory response and changes of the brain networks [71]. The next research performed on patients with VCI-ND hypothesized that enhanced glutamate neurotransmission might contribute to the preservation of cognitive functioning [72]. Also, Bella et al. [73] observed significant functional changes in intracortical excitatory neuronal circuits and clinical features in VCI-ND patients after rTMS treatment. Pan et al. [74] found that rTMS can improve cognitive function, especially regarding executive function, attention, memory, visuo-spatial abilities, and self-care ability, in patients with VCI-ND. Thus, it has high clinical application value. These changes indicated the role of rTMS in restoring the balance between the hemispheres’ activity and recovering cognitive function [75]. Cognitive function may be improved through rTMS by enhancing the metabolism of neuronal cells [70]. Above all, rTMS has been shown to significantly reduce serum lipid levels (cholesterol and triglycerides) [76] and to impact superoxide dismutase activity [77], which has been shown to be involved in influencing cognitive performance.

According to Padala et al. [64], Taylor et al. [67], Roque Roque et al. [68], and Durand et al. [58], the effects of rTMS on cognitive function in patients with MCI are related to the intensity of the stimulus, the frequency of the stimulation train, the site of stimulation, or even the course of treatment. These findings confirmed the evidence that rTMS might improve global cognitive function and, most of all, memory and executive functions in patients with MCI, having good acceptability and mild adverse effects [61,62]. Furthermore, some novel interventional targets, such as the precuneus, may be more effective therapeutic sites to improve memory in people with cognitive impairment [63,78]. Combining fMRI neuroimaging with rTMS could address causal relationships between task-related neural activation and cognitive performance [51,52]. Moreover, Yuan et al. [58] found that high-frequency rTMS can effectively enhance cognitive function in aMCI patients with altered spontaneous brain activity. In fact, high-frequency rTMS of the left DLPFC can be effective in alleviating cognitive symptoms in patients with MCI [56]. More recently, Esposito et al. [69] targeted the DLPFC with rTMS application, leading to a significant long-term increase in functional connectivity in MCI patients. Treatment by rTMS induced increased regional connectivity on the left DLPFC (i.e., the targeted area), also increasing the average distributed FC of the frontoparietal network [69]. It is worth highlighting that high-frequency rTMS has been shown to improve cognitive function, such as verbal fluency and memory [69,79,80]. High-frequency rTMS was applied to multiple coincident cortical sites associated with cognitive training, which may increase the probability of cortical plasticity [80,81]. Furthermore, high-frequency rTMS of the bilateral DLPFC has been shown to significantly enhance cognitive function, such as, respectively, executive performance and visuospatial function [82,83]. Particularly, rTMS at 10 Hz and 20 Hz has been shown to be more accurate and effective for attention [83] and executive functions [69,84]. Moreover, some evidence revealed that the settings of rTMS parameters (i.e., frequency, session number, stimulation site number) may significantly impact the effects on global cognitive function and that protocols with 10 Hz repetition frequency and stimulation of DLPFC for 20 sessions [69,84,85] can be able to produce long-term cognitive improvement in MCI. Further, pre-rTMS baseline activity and changes in the DMN at rest may be predictors of favorable rTMS treatment responses [17]. Whereas changes in functional connectivity within the DMN after targeted rTMS could represent a valuable indicator of treatment response [17].

## 5. Study Limitations

As in any systematic review, there is a possibility of missing additional articles because they were not found in our data-based search or because they were not available in the English language. Most of the publications that underwent full review were retrospective and could potentially have omitted relevant information. We did not include studies using intermittent theta-burst stimulation (iTBS), since this rTMS variant was applied to cohorts of patients with Alzheimer’s disease. This led to a lower chance of selection as our research focuses only on the MCI population. For this reason, we selected only seven articles, and one article described three case series (moderate quality and potential risk of bias). Another source of incomplete information was the calculation of the effect size by Cohen’s d (e.g., only the studies by Taylor et al. [67] and Roque Roque et al. [68] evaluated Cohen’s d). Moreover, no statistical analysis was conducted due to the limited number of included trials.

## 6. Conclusions

rTMS is a promising, non-invasive treatment for the improvement of cognitive function in elderly patients with cognitive impairment. Moreover, neuroimaging techniques in combination with rTMS have been shown to be useful for future network-based, fMRI-guided therapeutic approaches. To be specific, the combined application of rTMS with FC neuroimaging analyses and cognitive function assessments may help to clarify how rTMS exerts its effects on the human brain and would be useful to achieve more effective disease-modifying therapies for MCI patients.

## Figures and Tables

**Table 1:** Investigations details and patient demographics.

Study, Year	Country	Disease	N		M/F		Age		Type of Study
			Treatment	Sham	Treatment	Sham	Treatment	Sham	
Padala et al. [64], (2018)	United States	aMCI, naMCI	4	5	4/0	4/1	68.0 ± 10.0	64.0 ± 9.0	Double-blind, Randomized, Sham-controlled trial
Durand et al. [65], (2018)	France	aMCI, naMCI	3	N/A	1/2	N/A	69 ± 6.65	N/A	Three case studies
Cui et al. [66], (2019)	China	aMCI	21	N/A	N/A	N/A	50–85	50–85	Double-blind, Randomized, Sham-controlled trial
Taylor et al. [67], (2019)	United States	aMCI	66	33	N/A	N/A	55–90	55–90	Double-blind, Randomized, Sham-controlled, three-arm trial
Roque Roque et al. [68], (2021)	México	aMCI, naMCI	11	11	5/7	3/9	66.1 ± 5.5	67.2 ± 4.8	Randomized crossover trial
Yuan et al. [58], (2021)	China	aMCI	12	12	6/6	5/7	65.08 ± 4.89	64.67 ± 4.77	Double-blind, randomized, sham-controlled trial
Esposito et al. [69], (2022)	Italy	aMCI, naMCI	27	13	14/13	5/8	67.85 ± 9.28	66.77 ± 9.08	Double-blind, randomized, sham-controlled trial

Abbreviations: aMCI, amnestic mild cognitive impairment; naMCI, non-amnestic mild cognitive impairment.

**Table 2:** Summary of seven investigations that revealed potential benefits in cognitive functions after using rTMS in MCI.

Study, Year	Group	Intervention	Stimulation	Cognitive/Neuroimaging Findings	ORs, 95%Cis	Main Findings
Padala et al. [64], (2018)	Active r-TMS, sham-controlled	Non-navigated rTMS: 3000 pulses at 10 Hz, 4-s train duration, and 26-s inter-train interval, per session five times a week; % motor threshold: 120%	Left DLPFC	1- Apathy (AES-C)	*p <* 0.001	Significantly greater improvement in 3MS, MMSE, TMT-A, and CGI-I with rTMS compared to the sham treatment.
				2- Global cognition (3MS; global screen for cognition expanded from the MMSE)	*p <* 0.001	
				3- Executive function (TMT-A & TMT-B)	*p <* 0.05	
				4- Functional status (IADL)	*p* > 0.05	
				5- Patient’s global functioning (CGI-S, CGI I)	*p* > 0.05; *p <* 0.001	
				6- Caregiver burden (ZBS)	*p* > 0.05	
Durand et al. [65], (2018)	Active rTMS	The 3 patients received non-navigated rTMS (i.e., 10 Hz, 1 Hz, and 50 Hz-burst) sessions from 1 to 4 times a week; % motor threshold: 110%, 80%	Left/right DLPFC	1- Global cognition (MoCA)	N/A	The cognitive and clinical benefits of long-term rTMS treatment in MCI patients, without side effects, have been highlighted. This cognitive improvement is regardless of any anti-depressive effects.
				2- CGI-I	N/A	
				3- Depression (HDRS)	N/A	
Cui et al. [66], (2019)	Active rTMS, sham-controlled	Non-navigated rTMS: 30 trains of 5 s stimuli delivered at 10 Hz; 10-session daily treatment for about 2 weeks; % motor threshold: 90%	Right DLPFC	1- Global cognition (MMSE, ACE-III)	*p* < 0.001	rTMS-induced hypoconnectivity within DMN is associated with clinical cognitive improvements in patients with aMCI.
				2- Memory (Auditory Verbal Learning Test, AVLT, TMT-A & TMT-B)	*p <* 0.001	
				3- Geriatric Depression Scale (GDS)	*p* > 0.05	
				4- Functional connectivity (resting- state functional MRI)	*p <* 0.001	
Taylor et al. [67], (2019)	Active rTMS, sham-controlled	Navigated rTMS:10Hz delivers, 4000 pulses per session and up to 8000 pulses per day, with a total of 80,000 pulses over 2- to 4-week period; % motor threshold: 120%	Bilateral DLPFC Bilateral Lateral parietal cortex (LPC) Sham control	1- Memory (California Verbal Learning Test-II, CVLT-II)	*p* < 0.05	Positive effects of rTMS on cognitive and neuroimaging outcomes (i.e., global cognitive function, mood, and neuroimaging biomarkers).
				2- Global cognitive function (MoCA)	*p* < 0.05	
				3- Visuospatial episodic memory (BVMT-R)	*p* < 0.05	
				4- Language (BNT)	*p* < 0.05	
				5- Visuoconstructional function (ROCF)	*p* < 0.05	
				6- Speed of processing and executive control (TMT)	*p* < 0.05	
				7- Geriatric Depression Scale (GDS)	*p* < 0.05	
				8- Functional connectivity (resting state functional MRI)	*p* < 0.05	
Roque Roque et al. [68], (2021)	Active rTMS, sham-controlled	Non-navigated rTMS: 1500 pulses (30 trains of 50 pulses, each with a 10-s intertrain interval), at 5 Hz, for 30 sessions; % motor threshold: 100%	Left DLPFC	1- Global cognition (MoCA, MMSE)	*p* < 0.05	Statistically significant in the intergroup analysis with MoCA and intragroup only for the Active group.
				2- Mental health assessment (Mini-International Neuropsychiatric Interview, GDS)	*p* < 0.05	
				3- Neuropsychological assessment (NEUROPSI, ROCF, Stroop effect, and digit detection)	*p* < 0.05	
				4- Electroencephalographic (EEG) examination	N/A	
Yuan et al. [58], (2021)	Active rTMS, sham-controlled	Non-navigated rTMS: frequency of 10 Hz, five times per week over a period of 4 consecutive weeks; % motor threshold: 80%	Left DLPFC	1- Neuropsychological assessment (Clinical Dementia Rating Scale, Global Deterioration Scale, and MoCA)	*p* < 0.05	High-frequency rTMS can effectively improve cognitive function in aMCI patients and alter spontaneous brain activity.
				2- RS-fMRI (pre-processing and ALFF analysis)	*p* < 0.05	
Esposito et al. [69], (2022)	Active rTMS, sham-controlled	Non-navigated rTMS: frequency of 10 Hz, five times per week over a period of 4 consecutive weeks; % motor threshold: 80%	Bilateral DLPFC	1- Global cognition (RBANS)	*p* < 0.001	Significant long-term increase in FC in MCI patients in RS networks associated with executive functions.
				2- Beck Depression Inventory II	*p* > 0.05	
				3- Beck Anxiety Inventory	*p* > 0.05	
				4- AES	*p* ≤ 0.01	
				5- Functional connectivity (resting state functional MRI)	*p* < 0.001	

Abbreviations: MT, motor threshold; ACE-III, Addenbrooke’s cognitive examination; AES-C, Apathy Evaluation Scale clinician version; aMCI, amnestic mild cognitive impairment; CGI-I, clinical global impression—improvement; CGI-S—clinical global impression-severity; DLPFC, dorsolateral prefrontal cortex; FC, functional connectivity; GDS, Geriatric Depression Scale; IADL, instrumental activities of daily living; MINI, Mini-International Neuropsychiatric Interview; MMSE, Mini-Mental State Examination; MoCA, Montreal Cognitive Assessment; NEUROPSI, brief neuropsychological test battery; RBANS, Repeatable Battery for the Assessment of Neuropsychological Status; ROCF, Rey–Osterrieth complex figure; rTMS, repetitive transcranial magnetic stimulation; TMT-A and B, Trail Making Tests: A and B; ZBS, Zarit Burden Scale.

## Data Availability

Not applicable.
